# Wirelessly Powered Light and Temperature Sensors Facilitated by Electrically Small Omnidirectional and Huygens Dipole Antennas

**DOI:** 10.3390/s19091998

**Published:** 2019-04-29

**Authors:** Wei Lin, Richard W. Ziolkowski

**Affiliations:** Global Big Data Technologies Centre, School of Electrical and Data Engineering, Faculty of Engineering and IT, University of Technology Sydney, Ultimo, NSW 2007, Australia; richard.ziolkowski@uts.edu.au

**Keywords:** electrically small antennas, Internet of things (IoT), light sensors, rectennas, temperature sensors, wireless power transfer (WPT)

## Abstract

Wirelessly powered, very compact sensors are highly attractive for many emerging Internet-of-things (IoT) applications; they eliminate the need for on-board short-life and bulky batteries. In this study, two electrically small rectenna-based wirelessly powered light and temperature sensors were developed that operate at 915 MHz in the 902–928-MHz industrial, scientific, and medical (ISM) bands. First, a metamaterial-inspired near-field resonant parasitic (NFRP) Egyptian axe dipole (EAD) antenna was seamlessly integrated with a highly efficient sensor-augmented rectifier without any matching network. It was electrically small and very thin, and its omnidirectional property was ideal for capturing incident AC wireless power from any azimuthal direction and converting it into DC power. Both a photocell as the light sensor and a thermistor as the temperature sensor were demonstrated. The resistive properties of the photocell and thermistor changed the rectifier’s output voltage level; an acoustic alarm was activated once a threshold value was attained. Second, an electrically small, low-profile NFRP Huygens antenna was similarly integrated with the same light- and temperature-sensor-augmented rectifiers. Their unidirectional nature was very suitable for surface-mounted wireless power transfer (WPT) applications (i.e., on-body and on-wall sensors). Measurements of the prototypes of both the light- and temperature-sensor-augmented omni- and unidirectional rectenna systems confirmed their predicted performance characteristics.

## 1. Introduction

Wirelessly powered sensors are very attractive for many emerging Internet-of-things (IoT) applications, especially since the number of wireless IoT devices is expected to experience exponential growth in the upcoming 5G era [1,2,3,4,5]. For many sensor-based application scenarios, far-field wireless power transfer (WPT) technology is the most promising solution [6,7,8]. For instance, wireless sensors can be stationed on a tunnel wall or in a sealed storage room for detecting and monitoring critical parameters such as pressure, temperature, humidity, or illumination levels. Once such sensors have been installed, WPT avoids the need for replacing short-life batteries. Moreover, a WPT approach is more convenient and cost-effective for a sensor network since it enables charging multiple devices simultaneously. The rectenna (i.e., a receiving antenna integrated with a rectifier circuit) is the essential component of any far-field WPT system. It not only captures the AC electromagnetic waves, but it then converts them into DC power. Thus, it can power a sensor’s function, for example, turning on a buzzer in response to a certain level of light striking a photocell or in response to the temperature rising above some threshold. Furthermore, applications for which an on-board power source (e.g., a supercapacitor) is advantageous can also benefit from the WPT approach (i.e., by facilitating recharging it wirelessly and reducing space requirements).

**Figure 1:**
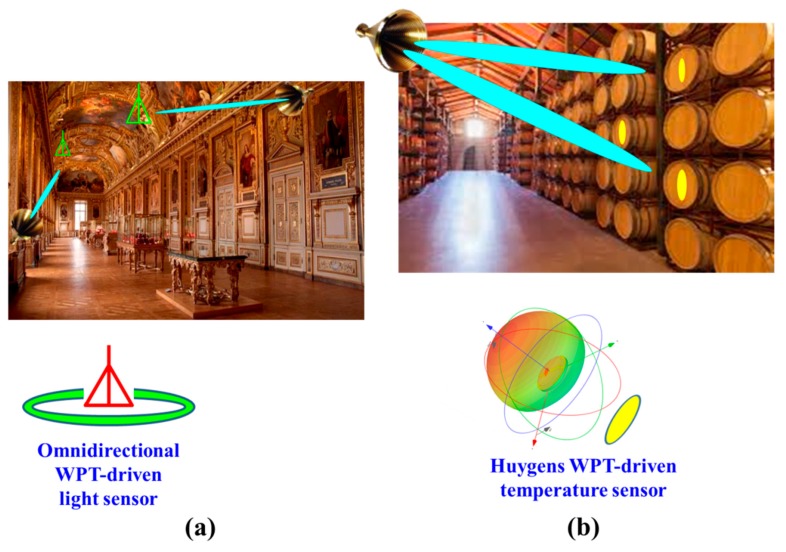
Illustrated applications of wirelessly powered Internet-of-things (IoT) sensors. (**a**) Ceiling light sensors in an art museum require omnidirectional wireless power transfer (WPT) capture capability. (**b**) Temperature sensors on wine barrels in a storage room require unidirectional broadside WPT capture capability.

Different wireless IoT application scenarios would require sensors powered by rectennas with a diverse set of wireless power capture characteristics. For example, a light, temperature, or humidity sensor could be placed on a tunnel’s ceiling and powered by radio frequency (RF) sources stationed at points along it. As illustrated in Figure 1a, this configuration would benefit from an omnidirectional rectenna. On the other hand, as illustrated in Figure 1b, similar light, temperature, or humidity sensors could be placed on the surface of each barrel in a wine cave. This configuration would benefit from a Huygens rectenna which would have a unidirectional pattern away from the surface on which it is placed. Furthermore, any potential IoT sensor would require its WPT rectenna to be compact in size, light weight, easy to fabricate, and readily integrated with it. Although many rectenna designs [9,10,11,12,13,14,15,16,17,18,19,20,21,22,23,24,25,26,27] have been investigated and reported in recent years, including the omnidirectional designs in [9,10,11,12,13,14,15] and the unidirectional designs in [16,17,18,19,20,21,22,23,24,25,26,27], they have not achieved highly compact, electrically small sizes (i.e., with their *ka* < 1, where *k* is the wave number at the operating frequency and *a* is the radius of the smallest sphere enclosing the entire rectenna), large wireless power capture capacities, and high AC-to-DC conversion efficiencies simultaneously.

In this article, temperature (thermistor) and light (photodiode) sensors that operate at 915 MHz in the 902–928-MHz industrial, scientific, and medical (ISM) bands are presented that are powered by both electrically small, highly efficient, metamaterial-inspired omnidirectional and Huygens dipole (unidirectional) rectennas. These rectennas were designed for their maximum WPT conversion efficiency. They have several advantages over previously reported systems. For example, the linearly polarized omnidirectional rectenna reported in [15] operates at the same frequency. However, it is much larger than the design presented here. The overall size is 120 × 120 mm, which means it is electrically large with its *ka* value being 1.66. The peak realized gain is 1.8 dBi and the maximum AC-to-DC conversion efficiency is only 75%. In comparison, the size of the linearly polarized omnidirectional rectenna reported herein is much smaller: 49.4 × 49.4 mm; hence, it is electrically small, with its *ka* being only 0.47. Even though its receive area is six times smaller in comparison, the realized gain is 1.75 dBi and the maximum AC-to-DC conversion efficiency is 87%. The temperature and light sensors are organically combined with these omni- and unidirectional rectennas to achieve very compact systems. 

First, a linearly polarized (LP) near-field resonant parasitic (NFRP) omnidirectional Egyptian axe dipole (EAD) rectenna was integrated with both a temperature- and a light-sensor-augmented rectifier circuit. It is a more advanced version of the previously reported NFRP rectenna in [28]. The entire system is highly compact (diameter is 0.13 *λ*_0_) and very thin (thickness is 0.002 *λ*_0_) with a *ka* value less than 0.5 (*ka* = 0.47). The highest gain of the EAD antenna is 1.75 dBi. The wireless power capture capacity of this EAD system is similar to a classic half-wavelength dipole rectenna, but its diameter is more than 3.8 times smaller. The simulated and measured characteristics of its optimized prototype are in good agreement.

Second, an advanced version of the electrically small Huygens dipole antenna concept [29] was similarly augmented to realize a wirelessly powered temperature and a light sensor with its unidirectional broadside–omnidirectional azimuthal pattern. This Huygens antenna system consists of a balanced pair of NFRP elements, the electric EAD, and the magnetic capacitively loaded loop (CLL). It is integrated with the same light- and temperature-sensor-augmented rectifier. It is electrically small (*ka* ≈ 0.73), is low profile (~0.04 *λ*_0_), has a large wireless power capture capacity (antenna gain is 3.8 dBi), and attains a very high (87.8%) conversion efficiency.

The performance characteristics of both the photocell-enabled sensors for light detection and the thermistor-enabled sensors for temperature assessment are discussed. The output DC voltage of the rectifier is controlled by the impedances of these sensors, which vary according to the conditions they are sensing. An acoustic device is connected to the output of the rectifiers as a threshold alarm for both the light and temperature levels. When the environment is too bright or the temperature is too high, the alarm will be activated. The developed highly compact wirelessly powered sensor systems are ideal candidates for quality control, security and hazard detection, and health monitoring applications, all of which are natural prospects for emerging wireless IoT systems. 

## 2. Electrically Small Omnidirectional WPT-Driven Light and Temperature Sensors

Electrically small, omnidirectional WPT-driven light and temperature sensors have been developed. They can monitor light conditions or ambient temperatures. These WPT-driven sensors consist of two primary parts: a receiving EAD antenna and a sensor-augmented rectifier circuit. Measured results from an optimized prototype validated their simulated performance characteristics.

### 2.1. Design of the Electrically Small EAD Antenna with Inductive Impedance

**Figure 2:**
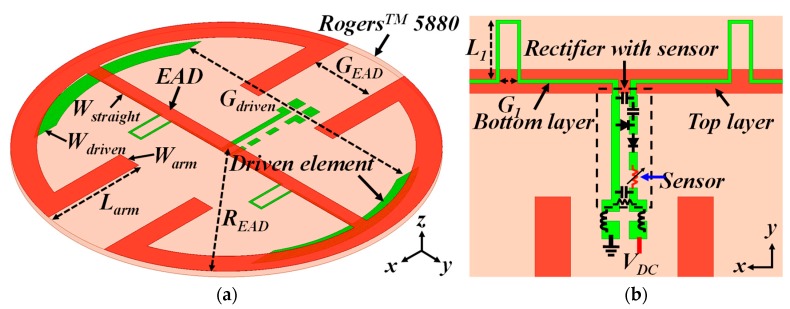
Electrically small (*ka* < 0.5) Egyptian axe dipole (EAD) antenna integrated with a sensor-augmented rectifier: (**a**) perspective view and (**b**) bottom view.

An electrically small EAD antenna with an inductive input impedance was designed to be directly matched to a highly capacitive rectifier circuit without any matching elements at 915 MHz in the corresponding ISM band and is illustrated in Figure 2. The entire design was etched on a disk of Taconic™ TLY copper-clad substrate, which had permittivity, permeability, and loss tangent values of 2.2, 1.0, and 0.0009, respectively, around 915 MHz, the operating frequency. The NFRP EAD element (in red color) was etched on the top metallization layer of the disk. Its four x-directed additional arms allowed it to be more compact and still attain the desired input impedance. In reception, it drove a short dipole that was connected directly to the rectifier (green color). The dipole and the sensor-augmented rectifier were etched on bottom layer of the disk. The dipole had two stubs that increased its overall length and, hence, facilitated attaining the desired inductive impedance. The rectifier circuit was seamlessly integrated to the dipole element in an orthogonal position and, hence, was electromagnetically isolated from it. The detailed parameters of the system were (in millimeters): *R_EAD_* = 24.7; *W_straight_* = 2.0; *W_arm_* = 3.0; *L_arm_* = 14.6; *G_EAD_* = 9.0; *L_driven_* = 44.4; *W_driven_* = 3.0; *G_driven_* = 35.2; *L*_1_ *=* 5.0; and *G*_1_ *=* 1.5. The entire system was highly compact (*ka* = 0.47) and easy to fabricate.

**Figure 3:**
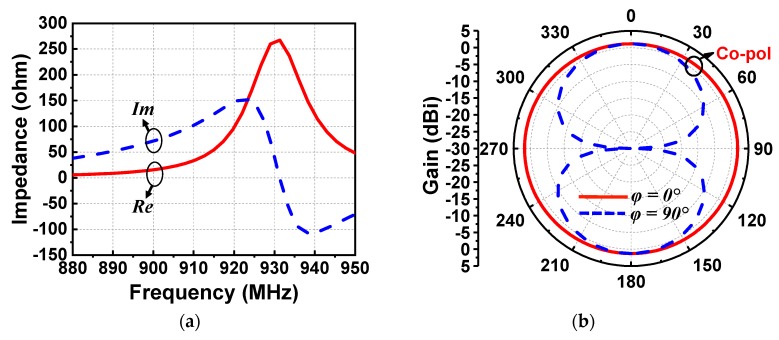
Simulated results of the EAD receiving antenna: (**a**) impedance and (**b**) gain patterns.

The EAD receiving antenna alone was simulated with the ANSYS Electromagnetics Suite 16.2. The input impedance and realized gain patterns are shown in Figure 3a,b, respectively. As desired, the reactance was inductive at the targeted 915 MHz. The input impedance was 54 + j124 Ω. Because it was an electrically small dipole antenna, it had the expected omnidirectional donut-shaped realized gain patterns. The peak realized gain was 1.75 dBi and the radiation efficiency was 87% at 915 MHz. It is noted that the diameter of this EAD design was more than 3.8 times smaller than a half-wavelength dipole antenna at that frequency. The radiation performance of the developed EAD antenna was excellent for its electrically small size. Measured patterns were not acquired because the antenna was not matched to the anechoic chamber’s 50-Ω source. Furthermore, it was demonstrated in [30] that the realized gain patterns of a 50-Ω version of this Huygens dipole antenna were in very good agreement with their simulated values.

### 2.2. Design of the Sensor-Augmented Rectifier Circuit 

**Figure 4:**
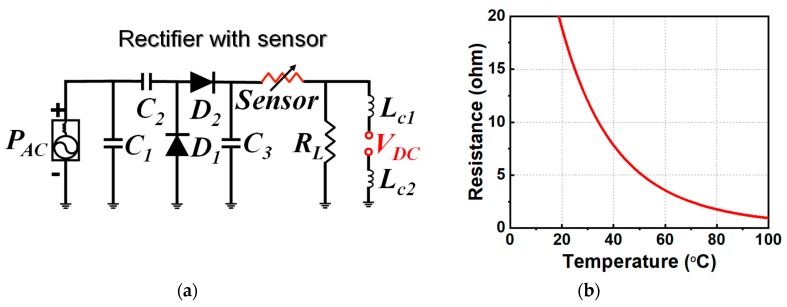
Sensor-augmented rectifier: (**a**) circuit model and (**b**) thermistor resistance as a function of the temperature.

The circuit model of the sensor-augmented rectifier is shown in Figure 4a. It consisted of three capacitors, two diodes, one impedance-dependent sensor, one load resistor, and two inductors that acted as RF chokes. From left to right, the essentially lossless capacitors *C*_1_ and *C*_2_ facilitated impedance matching. *C*_2_ also acted as an energy storage component during the negative portion of the sinusoidal AC signal. Two HSMS286C Schottky diodes, *D*_1_ and *D*_2_, were arranged to yield a voltage doubler. *C*_3_ acted as a low-pass filter and smoothed the output DC voltage. The sensor itself can be any impedance-dependent element, such as a photocell or thermistor. *R_L_* was the load resistor. *L_c_*_1_ and *L_c_*_2_ were the RF chokes. The detailed values of these components were: *C*_1_ = 0.4 pF; *C*_2_ = *C*_3_ = 100 pF; and *L_c_*_1_ = *L_c_*_2_ = 560 nH. The *R_L_* = 3.6 kΩ for light detection and 560 Ω for temperature sensing. 

The light detection was enabled with a photocell from Adafruit™ [31] that had a resistance around 1.5 kΩ in a bright environment and was almost open-circuited in a dark one. The temperature sensing was enabled with an negative temperature coefficient (NTC) thermistor from TDK™ [32]. Its resistance was a function of the temperature, as shown in Figure 4b. One can see that the resistance dropped quite quickly when the ambient temperature went up and, hence, the sensor was quite sensitive to temperature variations.

### 2.3. Experimental Results 

**Figure 5:**
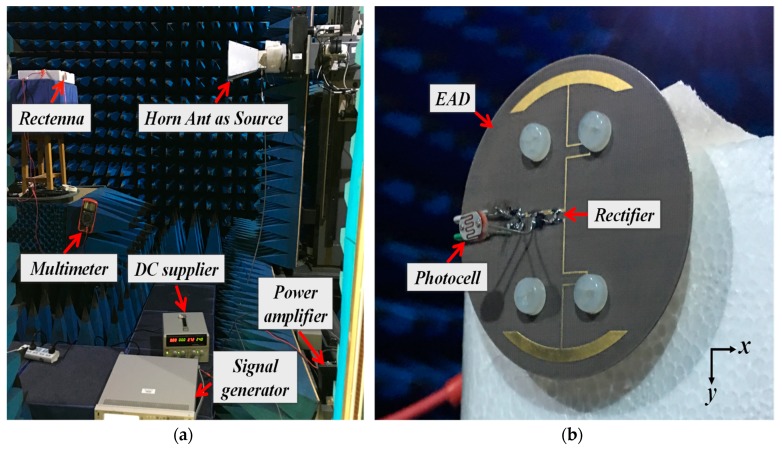
Sensor-augmented EAD rectenna: (**a**) experimental setup and (**b**) photocell-based prototype.

The optimized sensor-augmented rectenna was fabricated, assembled, and tested. The measurement setup was built in an anechoic chamber, as shown in Figure 5a. It included a signal generator, a power amplifier with DC supplier, a horn antenna, two coaxial cables, and a multimeter. The Keysight Technologies™ signal generator acted as the power source and provided the 915-MHz signal in the ISM band. A Mini-Circuit™ power amplifier magnified the signal which was delivered to a wideband (from 0.8 to 18 GHz) double-ridged horn antenna. This horn antenna radiated the electromagnetic waves to be received by the sensor system being tested. A multimeter measured the output DC voltage of the rectenna. The Friis transmission equation [33]

(1) $$\frac{P_{r}}{P_{t}}=G_{t}\times G_{r}\times {\left(\frac{\lambda_{0}}{4\pi R}\right)}^{2}$$

was used to calculate the power received by the rectenna $P_{r}$. This received power was readily determined since all of the requisite parameters were known. The realized gain of the horn antenna was *G_t_* = 7.0 dBi at 915 MHz (λ_0_ = 327.64 mm). The distance *R* between the horn antenna and the rectenna was 1.2 m, which was in the far-field region of the horn according to the standard criterion 2*D*^2^/*λ*_0_ [31]. The gain of the EAD antenna and the Huygens antenna below were known from the simulations. The received power *P_r_* was then determined according to the selected output power from the signal generator which defined *P_t_*. *P_t_* represents the power transmitted from the horn antenna. It was the net result of the output power of the signal generator, the gain of the power amplifier, and the losses incurred in the cables. 

Three sets of experiments were conducted. First, the output DC voltage and the AC-to-DC conversion efficiency of the rectenna with the 5.1-kΩ resistor load (without the sensor) were measured. Second, the output DC voltage values of the rectenna with the photocell present for different brightness levels were measured. An acoustic alarm from PUI Audio™ [34] was attached to the output port of the rectenna. It was turned on when the DC voltage exceeded 0.8 V. The experiments showed that the alarm would sound in a light environment but not in total darkness. The electrically small EAD rectenna with the photocell-augmented rectifier is shown in Figure 5b. Third, the output DC voltage values of the rectenna augmented with the thermistor were recorded at different ambient temperatures (i.e., the resistance of the thermistor changed with different temperatures, causing the output voltages to change). The alarm sounded when the temperature exceeded 65 °C.

**Figure 6:**
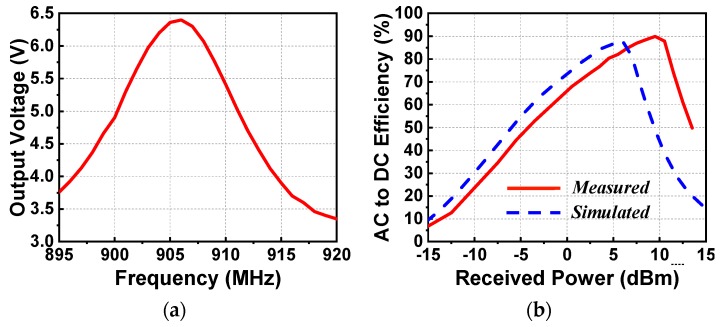
Measured results of the EAD rectenna. (**a**) Output DC voltage as a function of the source frequency. (**b**) Output DC voltage and AC-to-DC conversion efficiency as functions of the received wireless power.

In the first set of experiments, the output DC voltage as a function of the source frequency and the AC-to-DC conversion efficiency as a function of the received power were obtained. These results are shown in Figure 6. Figure 6a indicates that the peak output DC value was 6.4 V at 906 MHz when the received power was 9.5 dBm (when the output power of the signal generator was fixed to −13 dBm). The measured resonant frequency, 906 MHz, was only a 9-MHz shift (~1%) from its simulated value. Figure 6b compares the measured and simulation AC-to-DC conversion efficiencies as functions of the received power. The received power was calculated with the simulated gain of the EAD antenna being 1.27 dBi (*φ* = 0°, *θ* = 180°). The peak measured efficiency was 89.9%, which was very close to its simulated value, 87.8%.

To evaluate its omnidirectional wireless power capture capacity, the EAD rectenna was rotated at 45° steps around the *y*-axis, as shown in Figure 5b. The output DC voltage values were 6.4 V (*φ* = 0°, *θ* = 180°), 6.7 V (*φ* = 0°, *θ* = −90°), 6.4 V (*φ* = 0°, *θ* = 0°), and 6.7 V (*φ* = 0°, *θ* = 90°), respectively. Very good omnidirectional wireless power capture capacity was observed.

**Figure 7:**
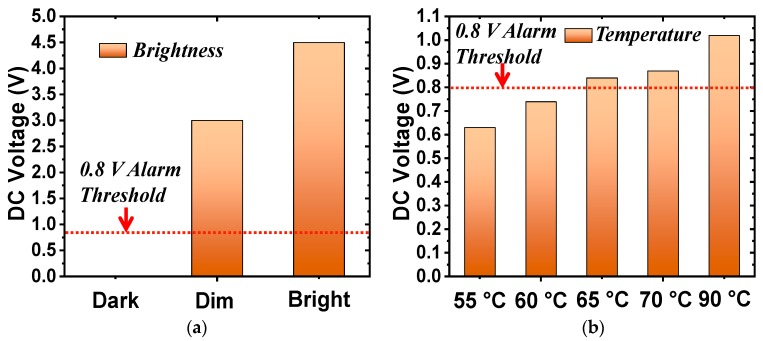
Measured output DC voltage of the sensor-augmented EAD rectennas. (**a**) Light sensor (photocell) system. (**b**) Temperature sensor (thermistor) system.

In the second set of measurements, the photocell augmented the rectifier. The load was replaced by the 3.6-kΩ resistor. The received power was fixed at 9.5 dBm. As shown in Figure 7a, the output DC voltage values were measured in three light intensity conditions. When the system was in total darkness, the resistance of the photocell was almost infinite (open-circuited) and, hence, the output DC voltage was zero. The resistance of the photocell dropped quickly under illumination, as shown in Figure 4b. The output DC voltage increased from 3.0 to 4.5 V, respectively, as the light changed from a dim to a bright environment. The alarm sounded when the DC voltage exceeded its 0.8-V threshold. Note that this threshold value could be adjusted by modifying the resistance of the alarm with additional lumped elements. The wirelessly powered light sensor was successfully demonstrated. It detected the different levels of light and notified the user when a specified threshold was exceeded. 

In the third set of the measurements, the thermistor replaced the photocell and the load resistance was changed to 560 Ω. Unfortunately, it was difficult to control the exact temperature in the anechoic chamber. Consequently, an alternative was adopted to mimic the resistance of the thermistor at different temperatures. Recall that the resistance of the thermistor as a function of the temperature is shown in Figure 4b. The resistance values were 4.3, 3.6, 3.0, 2.5, and 1.3 kΩ for 55, 60, 65, 70, and 90 °C, respectively. Resistors with these fixed-values were used to mimic the thermistor in the experiments. Figure 7b shows the output DC voltage values with the thermistor replaced with these equivalent resistors. The DC voltage exceeded the 0.8-V threshold above 65 °C, making the alarm sound. In contrast, the alarm did not turn on when the temperature was below 60 °C. Thus, the wirelessly powered temperature sensor was successfully demonstrated. It detected different temperatures and sounded the alarm once the temperature exceeded 65 °C. 

## 3. Electrically Small Huygens (Unidirectional, Broadside) Light and Temperature Sensors

Electrically small, light- and temperature-sensor-augmented Huygens rectennas have been developed. They are suitable, for example, for mounting on walls in a room for unidirectional power capture. As with their EAD counterparts, they can monitor light conditions or ambient temperatures. The Huygens rectenna design is based on those reported previously [35,36]. However, this is the first time that they have been equipped with sensors for light and temperature detection applications. Measured results from optimized prototypes validated their simulated performance characteristics.

### 3.1. Design of the Electrically Small Huygens Antenna with an Inductive Input Impedance

**Figure 8:**
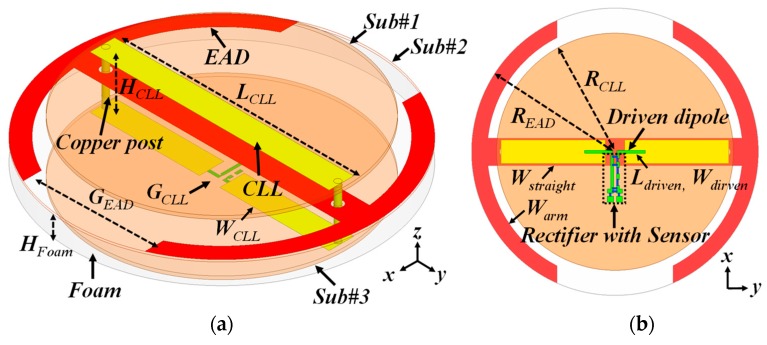
Electrically small (*ka* ≈ 0.73) Huygens antenna integrated with the sensor-augmented rectifier circuit: (**a**) perspective view and (**b**) bottom view.

The electrically small Huygens antenna design is shown in Figure 8. It organically integrates two metamaterial-inspired NFRP elements—an EAD and a CLL—to produce the Huygens pattern. Three disk-shaped PCB (Printed Circuit Board) substrates (Sub#1–#3) were selected to construct the system. The materials of Sub#1 and Sub#3 were Taconic™ TLY, which had permittivity, permeability, and loss tangent values of 2.2, 1, and 0.0009, respectively, around 915 MHz, the operating frequency. The thickness for both disks was 0.787 mm. The top and bottom parts of the CLL element were etched on the top surface of Sub#1 and Sub#3. They were then connected by two copper posts to form a loop. The material of Sub#2 was Rogers™ 5880, which had the same electrical properties as Taconic™ TLY, but its thickness was 0.508 mm. Sub#2 was located below Sub#1 and above Sub#3. The EAD was etched on the top surface of Sub#2. A short-dipole element was etched on the bottom layer of Sub#3 and was seamlessly integrated with the sensor-augmented rectifier circuit. A lossless Rohecell™ foam with the permittivity of 1.05 was used to support Sub#2. The detailed parameters of the system were (in millimeters): *R_EAD_* = 36.8; *W_straight_* = 7.0; *W_arm_* = 4.0; *G_EAD_* = 29.8; *R_CLL_* = 30.4; *L_CLL_* = 58.5; *H_CLL_* = 12.77; *W_CLL_* = 6.0; *G_CLL_* = 5.0; *L_driven_* = 15.56; *W_driven_* = 0.7; and *H_Foam_* = 7.5. The sensor-augmented rectifier was the same as that used in the EAD rectenna.

**Figure 9:**
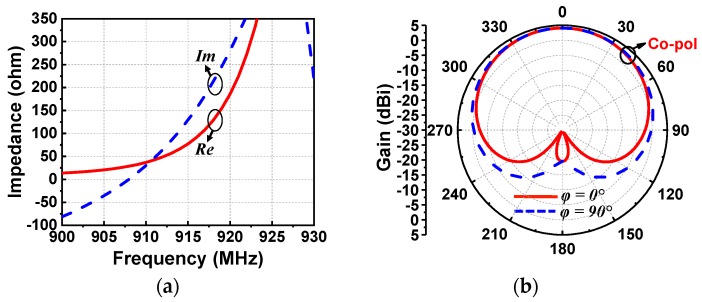
Simulated performance characteristics of the Huygens receiving antenna: (**a**) input impedance and (**b**) rain pattern.

As was achieved with the EAD rectenna, the Huygens rectenna was also designed to have an inductive impedance for direct matching to the rectifier. This was realized simply by adjusting the length of the dipole element. As shown in Figure 9a, the impedance was 77 + j129 Ω at 915 MHz, completely compensating for the capacitive reactance of the rectifier. As shown in Figure 9b, good Huygens radiation patterns were attained. These unidirectional patterns achieved a high realized gain value, 3.8 dBi, and a high FTBR (Front to Back Ratio) value, 23.5 dB. In contrast, the FTBR of the EAD system was near 0 dB. The simulated radiation efficiency was 80%. Again, measured patterns were not acquired for similar reasons. It was demonstrated in [35] that the realized gain patterns of a 50-Ω version of this Huygens dipole antenna were in very good agreement with their simulated values.

### 3.2. Experimental Results 

**Figure 10:**
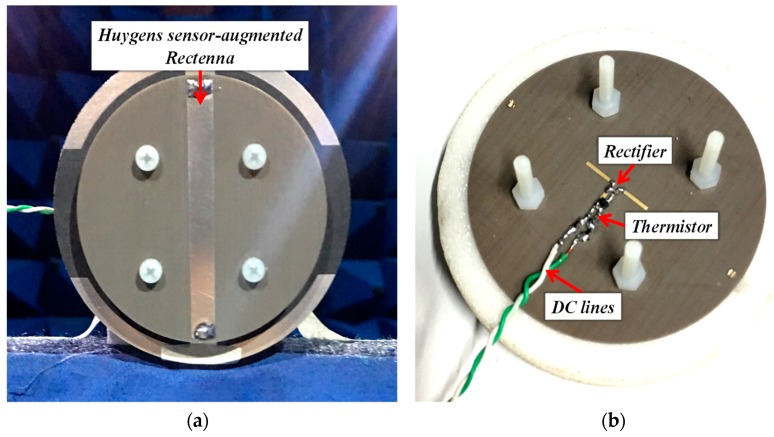
Fabricated and tested electrically small sensor-augmented Huygens rectenna: (**a**) front view and (**b**) bottom view.

The optimized sensor-augmented Huygens rectenna was fabricated, assembled, and tested. The prototype is shown in Figure 10a,b. It was compact (*ka* = 0.73) and of low profile (*H* = 0.04 *λ*_0_). The TDK™ thermistor with its SMD (Surface Mount Device) package was integrated into the rectifier, as shown in Figure 10b. As was done for the measurements of the corresponding EDA system, it was replaced by fixed value resistor values representing its response to specific temperatures. Three sets of experiments were conducted: the rectifier alone with the 5.1-kΩ resistor load (without sensor), the rectifier with the photocell and the 3.6-kΩ resistor load, and the rectifier with the resistors equivalent to the thermistor and the 560-Ω resistor load. 

**Figure 11:**
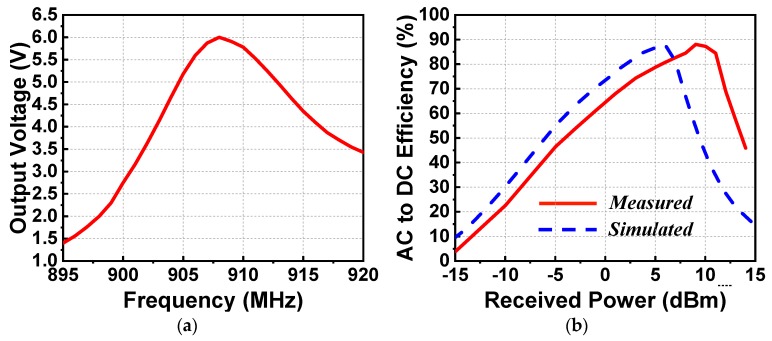
Measured results. (**a**) Output DC voltage as a function of the source frequency. (**b**) Output DC voltage and AC-to-DC conversion efficiency as functions of the received wireless power.

The output DC voltage values as functions of the source frequency and the AC-to-DC efficiency values as functions of the received power were measured in the first experiment. The results are shown in Figure 11. Because the broadside maximum gain of the Huygens antenna (3.8 dBi) was much higher than that of the EAD antenna (1.27 dBi for the tested configuration), the output power level of the signal generator was adjusted to −16 dBm. This decreased the power radiated by the horn, but the received power levels were maintained due to the increase of the gain associated with the receiving Huygens dipole antenna. As indicated in Figure 11a, the peak output voltage value was found to be 6.0 V at 908 MHz, which was shifted only 7 MHz (0.8%) from its simulated value of 915 MHz. Figure 11b shows the measured and simulated AC-to-DC efficiency. The measured peak efficiency was 88%, which was very close to the simulated value, 87.8%. This occurred when the received power was 9.0 dBm (the output power of the signal generator was fixed at −16 dBm). Consequently, the measured results demonstrated the unidirectional high wireless power capture capacity and high AC-to-DC conversion efficiency of the Huygens rectenna.

**Figure 12:**
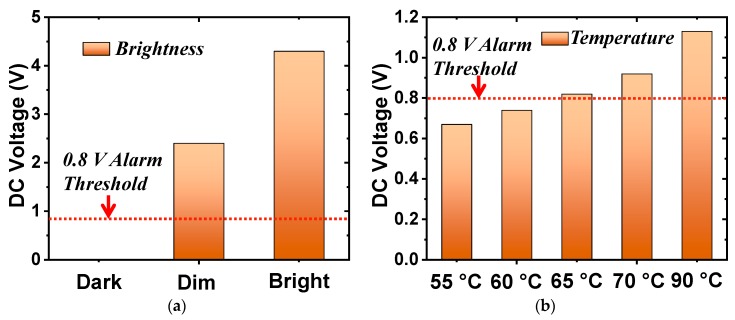
Measured output DC voltages of the sensor-augmented Huygens rectenna. (**a**) Light sensor (photocell) system. (**b**) Temperature sensor (thermistor) system.

The performance of the Huygens rectenna augmented with the photocell and the thermistor were also evaluated. The measured results are shown in Figure 12. The received power was set at 9.0 dBm for the maximum AC-to-DC conversion efficiency in both cases. The measured output DC voltages for the light detection system presented in Figure 12a exceeded the 0.8-V alarm threshold in the lit environments but did not for the dark one. The alarm was completely turned off in the dark case. The measured output DC voltages for the temperature-sensing system presented in Figure 12b exceeded the alarm threshold when the ambient temperature was higher than 65 °C. Both sets of measured results successfully verified the functionalities of both electrically small sensor-augmented Huygens rectenna systems. They are ideal candidates for wall-mounted wirelessly powered IoT sensors.

## 4. Conclusions

This article reported the investigations of both electrically small, highly compact sensor-augmented omnidirectional and Huygens (unidirectional) dipole rectennas for wirelessly powered IoT sensing applications. Both the EAD and Huygens rectennas performed as predicted. The experimental results demonstrated that both rectennas achieved high AC-to-DC conversion efficiencies (i.e., close to 90%). The EAD rectenna exhibited a large omnidirectional wireless power capture range. While the Huygens rectenna had a smaller (unidirectional) capture range, it had almost double the capture capability in that range (i.e., its cardioid pattern yielded a 3-dB realized gain enhancement). The PCB fabrication of both rectenna systems is straightforward and expedites their minimal cost levels. The EAD rectenna costs less than the Huygens rectenna simply because it only requires one PCB substrate. In both cases, the predicted performance characteristics of the light (photocell) and temperature (thermistor) sensor-augmented versions were successfully confirmed. The measured results demonstrated their effective light and temperature detection and the simultaneous ability for them to drive an alarm once a threshold voltage was exceeded. These wirelessly powered sensor systems illustrate the society-impactful potential for many emerging IoT wireless sensor-based applications.
